# Editorial: Children's Exercise Physiology

**DOI:** 10.3389/fphys.2020.00269

**Published:** 2020-04-15

**Authors:** Filipe Manuel Clemente, Luca Paolo Ardigò, Wook Song, Matthieu E. M. Lenoir, Luis Paulo Rodrigues, Hermundur Sigmundsson

**Affiliations:** ^1^Escola Superior Desporto e Lazer, Instituto Politécnico de Viana do Castelo, Rua Escola Industrial e Comercial de Nun'Álvares, Viana do Castelo, Portugal; ^2^Instituto de Telecomunicações, Delegação da Covilhã, Covilhã, Portugal; ^3^Department of Neurosciences, Biomedicine and Movement Sciences, School of Exercise and Sport Science, University of Verona, Verona, Italy; ^4^Institute of Sport Science, Seoul National University, Seoul, South Korea; ^5^Institute on Aging, Seoul National University, Seoul, South Korea; ^6^Department of Movement and Sports Sciences, Ghent University, Ghent, Belgium; ^7^Research Center in Sports Sciences, Health and Human Development, CIDESD, Vila Real, Portugal; ^8^Norwegian University of Science and Technology, Trondheim, Norway; ^9^Reykjavik University, Reykjavik, Iceland

**Keywords:** performance, physical fitness, measurement methodologies, motor competence, overweight and obesity, pathological subjects

Stimulated by the need to understand the specific effects of exercise on children, the current Frontiers Research Topic was carried out to collect a set of studies that highlight important findings related to the impact of exercise in this population. Childhood is a very specific and sensitive period for a great number of characteristics that are a part of human development. Among them, motor and functional changes, supported by growth (nature) and experience (nurture) play a key role in the performance trajectories of current and future development of children's physical fitness, motor competence, and physical activity behavior (Rodrigues et al., 2016) with relevance to future health profiles in adulthood (WHO, 2010; ODPHP, 2018). Exercise physiology research in this specific population has not always been a major concern, probably because maximal performance and competitive sports are not the intended targets in childhood; nonetheless, it is crucial to better understand children's aptitudes and to define exercise guidelines and optimization. That is why we expect that this Frontiers Research Topic on children's exercise physiology will help to boost the science and practice in childhood exercise and training.

With 20 articles published in this Research Topic, six main areas of research were defined: (a) performance, (b) physical fitness, (c) motor skill and fundamental motor competence, (d) measurement methodologies, (e) overweight subjects, and (f) pathological subjects. Most of the articles examined consider these areas of research.

Based on the diversity of study designs and objectives, we now have the opportunity to better understand the mechanisms that explain the effects of exercise on children and how performance and health can be mediated by different covariates.

It is not easy or straightforward to attribute an area to each article published in our Research Topic, though we have tried to do so. We have also summarized the most noteworthy evidence of each study.

## Performance

Using a cross-sectional design and focusing on analyzing weightlifting performance from 2013 to 2017 in the World Championships and Olympic Games, a study conducted by Huebner and Perperoglou aimed to estimate the age of peak performance and quantity performance development during the transition from adolescence to adulthood.

By following the characterization of anthropometric variables and adding the maturity information of handball players, Hammami et al. aimed to determine the importance of such variables on the fitness status of players from under-14 to under-18 age groups. The findings reveal that age was the strongest predictor of performance (e.g., jumping, sprinting, change of direction, and strength), and that only body mass contributed to the prediction of jumping ability (Hammami et al.).

Also within the topic of performance—and considering the hypothesis that physical activity and fitness status can be associated with school achievements—Padulo et al. suggests that agility is moderately associated with academic achievement in English (*r* = −0.400), Italian (*r* = −0.337), maths (*r* = −0.423), music (*r* = −0.315), sports (*r* = −0.622), and technology (*r* = −0.381). In the same study (Padulo et al.), it was found that lifestyle and socio-demographics significantly impacted school achievements. Specifically, lifestyle fully moderated the impact of family context on academic achievement.

Finally, a study conducted on under-14 soccer players tested the effects of multidirectional plyometric training on vertical jump height, change of direction, and dynamic postural control (Jlid et al.). After an 8-week training period (2 days per week), meaningful improvements were found for squat jump (+11.14%), countermovement jump (9.91%), and *t*-test (−3.07%) in the experimental group, while no significant changes were observed in the control group.

## Physical Fitness

A study involving a sample of 18,295 schoolchildren was conducted to test the possibility of aerobic capacity related to other aspects of health-related physical fitness (Venckunas et al.). According to the results, aerobic capacity was most strongly related to agility shuttle run and standing broad jump, which explained more than 10% of the variance in players' performance in these tests (Venckunas et al.). Aerobic capacity also explained 6 to 7% of performance in terms of abdominal curls and bent arm hang time. The positive contribution of aerobic capacity was revealed for both sexes and all age groups (Venckunas et al.).

Sánchez-Sánchez et al. investigated the influence of age group on athletic performance and muscle response after a repeated sprint ability test. The differences between the first and the last sprint, as well as the percentage of decrease, between age groups were insignificant (Sánchez-Sánchez et al.). The musculo-mechanical properties of participants in the under-16 and under-18 age-groups changed after repeated sprints.

Kokstejn et al. tested whether fundamental motor skills contribute to the acquisition of soccer-specific motor skills while considering the physical fitness and biological maturation of young soccer players. The linear regression results reveal that fundamental motor skills and physical fitness were significant predictors of speed dribbling performance, although only the effect of fundamental motor skills was statistically significant (Kokstejn et al.).

Branco et al. tested the effects of two-concurrent training programs conducted over 12 weeks in obese children. They found significant increases in musculoskeletal mass and resting metabolic rate, significant reductions in fat mass and body fat percentage, significant improvements in maximum isometric handgrip strength, maximum isometric lumbar-traction strength, maximum isometric lower-body strength, and maximal oxygen consumption, and significant reductions in insulin, homeostatic model assessments, triglycerides, total cholesterol, and low-density lipoprotein.

A mini-review written by Armstrong and Welsman highlights the importance of properly understanding the results of peak oxygen uptake and emphasizes some flaws and fallacious interpretations of the peak oxygen uptake ratio with regard to body mass. It has been demonstrated that peak oxygen uptake increases in accordance with sex-specific, concurrent changes in age- and maturity-status-driven morphological covariates (Armstrong and Welsman). Interestingly, fat-free mass has been suggested as the most powerful morphological variable in terms of its impact on the development of youth aerobic fitness considering cycle ergometry and treadmill running tests (Armstrong and Welsman). Finally, the mini-review also recommends that future cross-sectional studies should consider sex-specific and maturation-driven changes in fat-free mass as covariate factors.

## Measurement Methodologies

Forbregd et al. assessed the effects of four different exercise modalities and body positions—treadmill walking/running (modified Bruce protocol), sitting, tilted (45 degrees), and lying flat ergometer bicycling (viz., with patient less in-motion)—on peak oxygen uptake (VO_2_), stroke volume, heart rate, and cardiac output in 31 9 to 15-year-old children under cardiopulmonary exercise testing (CPET). When compared with participants who completed the treadmill test, those who performed bicycling elicited lower peak VO_2_s. Peak heart rate decreased from both treadmill to upright bicycle and from upright bicycle to lying flat bicycle. Overall, considering the higher VO_2_ with treadmill testing, both sitting and lying flat bicycling tests are judged proper for CPET with concomitant MRI-scanning, PET-scanning, and echocardiography.

Gao et al. investigated differences between sedentary and non-sedentary activities in 35 7 to 11-year-old children (21 girls) in terms of VO_2_, triaxial accelerometry, and thigh muscle electromyography (EMG) during eight different energy-demanding activities: lying down supine while watching a children's program, sitting quietly and playing a mobile game, standing quietly and playing the mobile game, walking on a treadmill at either 4 or 6 km/h, and walking around an indoor track at freely chosen speed (5.0 ± 0.8 km/h). Optimal sedentary-to-non-sedentary thresholds, based on a receiver operating characteristic (ROC) curve analysis, revealed values of 1.3 for METs (sensitivity = 82%, specificity = 88%), 0.0033 g for accelerometry mean amplitude deviation (sensitivity = 80%, specificity = 91%), and 11.9% for EMG (sensitivity = 79%, specificity = 92%).

Duncan et al. used VO_2_ and triaxial accelerometry to assess physical activity in 30 8 to 11-year-old children (14 girls) while lying down supine, playing with Lego, walking on a treadmill at either 4.5 or 6.5 km/h, running on a treadmill at 6.5 km/h, overarm throwing and catching, instep passing a football, and cycling (35 W). Participants wore accelerometers on their non-dominant wrist, dominant wrist, waist, and ankle. Based on VO_2_ values, lying down supine and playing with Lego were categorized as sedentary activities (<1.5 METs), walking and throwing and catching were light activities (1.51–2.99 METs), and running, cycling, and instep passing were moderate activities (>3 METs). According to the ROC curve analysis, sedentary-to-non-sedentary and sedentary-to-moderately-non-sedentary activity discrimination were excellent for all accelerometer placements (with the best results being associated with the ankle accelerometer), even when cycling was neglected.

The protocol article by Petraconi et al. describes a child-friendly Go/No-Go paradigm to assess inhibitory control of the foot with a physical activity measurement methodology based on a dance mat, tested in 31 3 to 4-year-old children (17 girls). Go and No-Go stimuli were modeled within the context of a fishing game, and children's behavioral responses were assessed by recording the latency to touch the mat and the accuracy of the touches. The dance mat protocol can be used by researchers who are interested in the development of foot motor response inhibition in young children, including hand and foot specialization, exercise, and/or sport performance, and in neuroimaging (i.e., with fNIRS).

## Motor Competence

Coppens et al. followed the development of the motor competence of over 550 children over 2 years. Gender, BMI, age, and physical fitness measures (speed and explosivity) were cross-sectionally related to motor competence at the baseline, which is in line with findings presented in the literature published in past decades. From a longitudinal perspective, girls made less progress than boys in terms of motor competence during the 2-year study. Surprisingly, though, apart from BMI—which was negatively associated with MC development—none of the physical fitness measures affected the rate of development.

A study conducted by Hill et al. provides new insights into the development of a crucial component of motor competence, namely, dynamic postural control. Specifically, they studied the contribution of the upper extremities to the performance of three dynamic balance tests that were performed with free and restricted arm movements. Arm movements were clearly beneficial to performance on the Y-balance test and on a dynamic walking test. However, when participants were made to regain their stability on one leg after the execution of a jump, this advantage did not emerge. Apparently, the neuromuscular response strategies of the ankle, knee, and hip might be sufficient to effectively guide the transition from dynamic to static balance. This study sheds light on the underexplored role of the arms in maintaining postural balance and, therefore, might have implications for training and therapy.

Physical performance measures are assumed to support the development of general motor competence. González-Víllora et al. compared two methods of small-sided futsal games to improve physical (e.g., distance covered during the game) and physiological (e.g., heart rate) variables in elementary school children. They showed that the Contextualized Sport Alphabetization Model (CSAM; Kirk, 2017) was associated with higher scores regarding the physical and physiological aspects of small-sided futsal games when compared to the well-known Teaching Games for Understanding (TGfU) approach. The relevance of this study lies in underlining the value of the CSAM model for introduction in small-sided games, focusing on the development of cognition, technique, and social skills, while respecting the necessity that PE sessions at school should promote optimal involvement at the physiological level of each pupil.

Along similar lines of thinking, Garcia-Angulo et al. used principles of non-linear pedagogy by modifying games so that the level of play suited the children's developmental characteristics. The impact of changing the task and environment of the physical activity levels during the game was studied by measuring heart rate in young players between 6 and 12 years of age. The results show that these games led to a physical activity rate that meets the international PA recommendations favoring health benefits.

The quest to identify young athletes with the potential to excel in a particular sport is prominent in the scientific literature. Zhao et al. applied a test battery of 24 non-sport-specific tests on a squad of under-15 youth elite athletes in six different sports. The proposed test battery showed a medium-to-high validity for discriminating between basketball, fencing, judo, swimming, table tennis, and volleyball. In addition, the tests could discriminate between athletes from one sport and those from the other five sports with an acceptable level of accuracy.

Carvalho et al. studied the evolution of the physiological performance characteristics of youth female basketball players during the pubertal years. They discuss their findings with respect to calendar age (CA), biological age (BA, based upon age of menarche), and sports age (SA, exposure to training). They showed that the developmental rate of explosivity and speed/agility starts to level off at around the age of 14, although the models keep increasing linearly until 3 years after the age of menarche. Contrarily, a linear trend of improvement is observed when endurance and the overall performance index are aligned for CA and BA. It was observed that when the effects of maturation reach their end, all girls evolve in a similar way.

## Overweight and Obesity

Given the many health-related correlates of overweight and obesity, the availability of reference values and validated cut-offs in different populations is crucial. In their study, López-Sánchez et al. assessed the prevalence of overweight and obesity in a sample of 1,000 children and adolescents in Italy and Spain. Overweight or obese status was determined by means of three different international references: BMI, according to the World Health Organization (WHO) International Obesity Task Force (IOTF), and fat mass (FM), according to the Child Growth Foundation (CGF). The three classifications produced different levels of prevalence of overweight and obesity.

Overweight and obesity are generally considered obstacles to participation in physical activities and a healthy lifestyle. Pojskic and Eslami focused on the interrelationship between age, indices of overweight/obesity, physical activity, and cardiorespiratory fitness (CRF) in a sample of >750 children and adolescents in Bosnia and Herzegovina. Overall, CRF was associated with gender, age, indices of overweight/obesity, and PA levels. A worrisome finding was that over 80% of the participants were categorized as having low CRF, which puts them at risk for metabolic diseases. At the fundamental level, overweight status was not associated directly with CRF. Rather, old male participants with high levels of PA exhibited better measures of CRF than other participants.

While overweight and obesity are known to affect movement control and efficiency in tasks that demand the movement of large parts of the body, relatively little is known about how body fat percentage is related to mechanical efficiency during cycling at different intensities and how this might be associated with hormonal responses to exercise. To this end, Jabbour and Majed had male adolescents perform an incremental exercise test to exhaustion while measuring energy consumption, lipid oxidation rate, and concentrations of epinephrine and norepinephrine. As expected, mechanical efficiency decreased as weight increased. Interestingly, the authors show that the mechanisms of lower ME vary with intensity. At a low intensity, overweight and obese boys exhibited increased energy consumption, leading to lower ME. Meanwhile, at a higher intensity, lower ME is primarily explained by low power output and hormonal responses to exercise.

Sögüt et al. also compared central adiposity, cardiovascular fitness, and physical activity in children, revealing that moderate to vigorous levels of physical activity were negatively associated with body mass index and waist circumference and positively correlated with cardiovascular fitness in both sexes.

## Pathological Subjects

A study conducted by Pianosi and Smith dealt with the ventilatory limitation of exercise in children with exertional dyspnea. The traditional method for evaluating ventilatory limitation considers breathing reserve (Pianosi and Smith). However, this approach is problematic in that it ignores maximal voluntary ventilation (MVV). Therefore, Pianosi and Smith used multiples of the FEV_1_ method. Their results reveals that evaluating ventilatory limitations in children using 30FEV_1_ is superior to using MVV-based methods.

A study conducted by Rochette et al. measured the fat and carbohydrate oxidation rates of children with inactive JIA and healthy children (control group) using a submaximal incremental exercise test. The results show lower lipid oxidation rates and higher respiratory exchange ratio at 50% of VO_2_ peak in the JIA group in comparison to the control group. However, there were no differences in heart rate or percentage of VO_2_ peak at the maximal fat oxidation rate (MFO), and healthy subjects reached their MFO at a higher exercise power than JIA subjects did Rochette et al.

Regarding metabolic-related diseases, Dring et al. examined the risk factors for cardio-metabolic disease [e.g., multi-stage fitness test (MSFT), VO_2_ peak, and adiposity] in children. Children were separated into quartiles based on the distance run in the MSFT. The worst-performing group had the highest blood IL-6 (3.25 ± 0.25 pg/mL) and IL-1β (4.78 ± 0.54 pg/mL) levels and the lowest concentrations of IL-10 (1.80 ± 0.27 pg/mL).

Cystic fibrosis (CF) is a serious genetic disease that typically affects the lungs and seriously reduces exercise performance (Shei et al.). In a review article authored by Shei et al. it is stated that few studies have provided evidence for physiological differences in CF related to exercise capacity through aging. Therefore, they recommend that exercise studies in CF should consider factors like pulmonary function declination, chronic airway colonization, endocrine comorbidities, and nutrition-related factors because these are age-specific factors that affect the exercise capacity of CF patients.

A study comparing 16 children after coarctation repair and 20 healthy control subjects revealed that children after coarctation repair exhibited meaningfully lower peak power and maximal oxygen uptake than healthy children (Vandekerckhove et al.). The amount of muscle tissue oxygenated was also meaningfully lower in patients (from 10 to 70% peak power output), and muscle deoxygenated hemoglobin was significantly higher in patients (from 20 to 80% peak power output).

## Author Contributions

All authors listed have made a substantial, direct and intellectual contribution to the work, and approved it for publication.

### Conflict of Interest

The authors declare that the research was conducted in the absence of any commercial or financial relationships that could be construed as a potential conflict of interest.
